# Multicentre quantitative ^68^Ga PET/CT performance harmonisation

**DOI:** 10.1186/s40658-019-0253-z

**Published:** 2019-11-08

**Authors:** Daphne M. V. Huizing, Daniëlle Koopman, Jorn A. van Dalen, Martin Gotthardt, Ronald Boellaard, Terez Sera, Michiel Sinaasappel, Marcel P. M. Stokkel, Berlinda J. de Wit-van der Veen

**Affiliations:** 1 Department of Nuclear Medicine, Netherlands Cancer Institute, Amsterdam, The Netherlands; 2 Department of Nuclear Medicine, Isala, Zwolle, The Netherlands; 3 Department of Medical Physics, Isala, Zwolle, The Netherlands; 4 Department of Radiology and Nuclear Medicine, Radboud University Medical Center, Nijmegen, The Netherlands; 5 Department of Radiology and Nuclear Medicine, Amsterdam University Medical Centres, location VU University Medical Center, Amsterdam, The Netherlands; 6 Department of Nuclear Medicine and Molecular Imaging, University of Groningen and University Medical Center Groningen, Groningen, The Netherlands; 7 EANM Research Limited (EARL), Vienna, Austria; 8 Department of Physics, Netherlands Cancer Institute, Amsterdam, The Netherlands

**Keywords:** Quantification, ^68^Gallium PET/CT, Image quality, Harmonisation

## Abstract

**Purpose:**

Performance standards for quantitative ^18^F-FDG PET/CT studies are provided by the EANM Research Ltd. (EARL) to enable comparability of quantitative PET in multicentre studies. Yet, such specifications are not available for ^68^Ga. Therefore, our aim was to evaluate ^68^Ga-PET/CT quantification variability in a multicentre setting.

**Methods:**

A survey across Dutch hospitals was performed to evaluate differences in clinical ^68^Ga PET/CT study protocols. ^68^Ga and ^18^F phantom acquisitions were performed by 8 centres with 13 different PET/CT systems according to EARL protocol. The cylindrical phantom and NEMA image quality (IQ) phantom were used to assess image noise and to identify recovery coefficients (RCs) for quantitative analysis. Both phantoms were used to evaluate cross-calibration between the PET/CT system and local dose calibrator.

**Results:**

The survey across Dutch hospitals showed a large variation in clinical ^68^Ga PET/CT acquisition and reconstruction protocols. ^68^Ga PET/CT image noise was below 10%. Cross-calibration was within 10% deviation, except for one system to overestimate ^18^F and two systems to underestimate the ^68^Ga activity concentration. RC-curves for ^18^F and ^68^Ga were within and on the lower limit of current EARL standards, respectively. After correction for local ^68^Ga/^18^F cross-calibration, mean ^68^Ga performance was 5% below mean EARL performance specifications.

**Conclusions:**

^68^Ga PET/CT quantification performs on the lower limits of the current EARL RC standards for ^18^F. Correction for local ^68^Ga/^18^F cross-calibration mismatch is advised, while maintaining the EARL reconstruction protocol thereby avoiding multiple EARL protocols.

## Introduction

The use of ^68^Gallium (^68^Ga)-labelled peptides for PET imaging has increased in the past years with the market authorisation for ^68^Ga/^68^Ge-generators. The main applications include imaging of neuroendocrine tumours using somatostatin analogues and prostate cancer imaging using the prostate-specific membrane antigen [1, 2]. Though the interpretation of ^68^Ga-PET/CT is mainly based on visual assessment, quantitative measures should be used to evaluate or predict therapy response.

Previous experience with ^18^Fluorine (^18^F) expressed the need for standardisation of acquisition and reconstruction protocols in order to retrieve comparable quantitative imaging data. The EANM Research Ltd. (EARL) provides an accreditation programme to ensure PET/CT system harmonisation in multicentre ^18^F-FDG PET/CT studies [3]. This approach is based on standardizing the recovery coefficient (RC) for six phantom spheres with different sizes, thereby minimising inter- and intra-institute variability. For other isotopes, quantification should be evaluated separately as isotope characteristics can result in different image quality and quantification accuracy. For example, Makris et al. studied ^89^Zirconium (^89^Zr) PET and showed the need for a specific harmonisation step including post-reconstruction smoothing to enable comparable quantitative measures among PET/CT systems [4]. In contrast, a recent ^18^F performance study showed that post-reconstruction filtering is not required for state-of-the-art PET/CT systems in relation to this isotope [5]. However, for ^68^Ga, such studies are not yet available.

In general, PET quantification accuracy depends on reconstructions, noise, and spatial resolution [6]. For ^68^Ga, the lower positron yield (89%), long positron range due to high initial positron energy (max 1.90 MeV, mean 0.84 MeV), short physical half-life (68 min) and small prompt gamma branching (3.2%, 1.077 MeV) may result in an inferior image quality compared to ^18^F [7]. Therefore, the aim of this study was to assess ^68^Ga-PET/CT quantification accuracy and reproducibility in a multicentre setting based on EARL standards.

## Materials and methods

### Clinical protocol evaluation

A survey among eight Dutch hospitals was performed to evaluate factors that affect quantification and to assess variability in clinical ^68^Ga-PET/CT acquisition protocols. Questions focussed on administered activity, PET/CT system, and acquisition- and reconstruction settings.

### ^18^F and ^68^Ga PET/CT phantom acquisitions

Eight European hospitals with 13 PET/CT systems performed phantom acquisitions, of which 11 systems were EARL accredited, but all had recoveries within the published EARL specifications. Six Biograph mCT systems (Siemens Healthineers, Erlangen, Germany), three Discovery systems (GE Healthcare, Milwaukee, WI, USA) and four Philips systems (Philips Healthcare, Eindhoven, The Netherlands) were included.

^18^F and ^68^Ga acquisitions were performed at the end of 2017 and beginning of 2018 with two phantoms which were prepared using a standardised procedure by experienced staff from each centre. First, the NEMA PET cylindrical phantom was filled with 6–13 kBq/ml of ^18^F and ^68^Ga. Second, the NEMA NU-2 Image Quality (IQ) phantom was imaged using a 1:10 ratio with 2.0 and 20.0 kBq/ml of ^18^F and ^68^Ga in background compartment and spheres (37, 28, 21, 17, 13, and 10 mm diameter), respectively. Acquisitions of both phantoms were performed with minimal two bed positions and at least 5 min per bed position. Images were reconstructed according to local settings, including corrections for decay, randoms, dead time, CT-based attenuation, and scatter.

### Data analysis

Image noise was characterized for ^68^Ga only using the coefficient of variation (CoV) along a 30 × 30 × 160 mm bar in the centre of the cylindrical phantom. Image quality was based on the RC of all six spheres, analysed by the EARL semi-automatic tool [5, 8]. The RC_max_, RC_peak_ and RC_mean_ were determined as a function of sphere size based on the maximum voxel value (RC_max_), the 1.0 cm^3^ volume with the maximised average value (RC_peak_) and the mean value of 50% isocontour of the maximum voxel value (RC_mean_) with contrast correction, respectively. A spherical volume-of-interest (VOI) of ~ 300 ml in the centre of the cylindrical phantom and ten VOIs in the background of the IQ phantom were used for local PET and dose calibrator cross-calibration. IQ phantom background volume was 9400 ml, unless specified otherwise by the institute.

## Results

Eight Dutch hospitals provided their clinical acquisition- and reconstruction protocols (Table 1), which showed to be different.

**Table 1:** Acquisition and reconstruction settings of clinical ^68^Ga PET/CT imaging for prostate cancer and neuroendocrine tumours. One hospital per row is presented

Site	PET/CT system	Reconstruction settings	Prostate cancer			Neuroendocrine tumours		
			Minutes per bed position		Injected activity	Minutes per bed position		Injected activity
A	Philips Gemini TOF 64	BLOB-OS-TF 4 mm 3i33ss	Pelvis: 4	Body: 3	1.5 MBq/kg (range 50–250 MBq)	< 90 kg: 2.5	> 90 kg: 3.5	2.6 MBq/kg (range 100–160 MBq)
B	Philips Gemini TF and XL	Astonish iterative reconstruction	4		2.0 MBq/kg	4		2.6 MBq/kg
C	Siemens mCT Flow	TrueX + TOF 2i21ss Gaussian 5mm	1.5 mm/s CTM		2.0 MBq/kg	2.5		100 MBq
D	Philips Ingenuity TF	BLOB-OS-TF 4 mm 3i33ss 2 mm smooth B filter	NA			4		< 90 kg: 150 MBq > 90 kg: 200 MBq
E	Siemens mCT TrueV	OSEM3D, TOF + PSF 2i21ss Gaussian 5 mm	4		1.5 MBq/kg (min 80 MBq)	NA		
F	Philips Gemini TOF	BLOB-OS-TF 4 mm 3i33ss	Pelvis: 3	Body: 2	100 MBq	2.5		100 MBq
G	Siemens mCT	TrueX + TOF 4i21ss Gaussian 5 mm	3		1.5 MBq/kg	3		1.5 MBq/kg
H	Siemens mCT40 and mCT128	TrueX + TOF 3i21ss Gaussian 3 mm	< 70 kg: 1.5 MBq/kg: 3 1.13 MBq/ml: 4 0.9 MBq/ml: 5	> 70 kg: 1.5 MBq/kg: 4 1.2 MBq/ml: 5 1 MBq/ml: 6	1.5 MBq/kg	< 70 kg: 1.5 MBq/kg: 3 1.13 MBq/ml: 4 0.9 MBq/ml: 5	> 70 kg: 1.5 MBq/kg: 4 1.2 MBq/ml: 5 1 MBq/ml: 6	1.5 MBq/kg

*NA* = not applicable, *i* = iteration, *ss* = subsets, *TOF* = time-of-flight, *PSF* = point-spread-function, *CTM* = continuous table motion

**Fig. 1:**
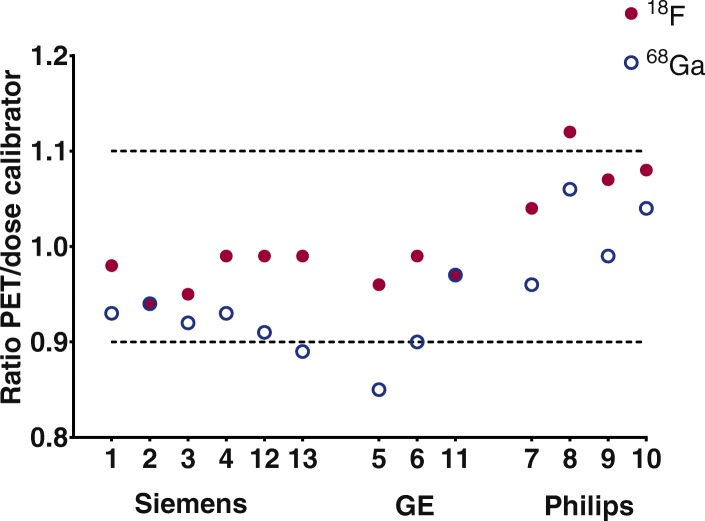
Accuracy of the measured activity by the PET/CT system and local dose calibrator, based on the average between the cylindrical and IQ phantom. Numbers correspond to Table 2

**Fig. 2:**
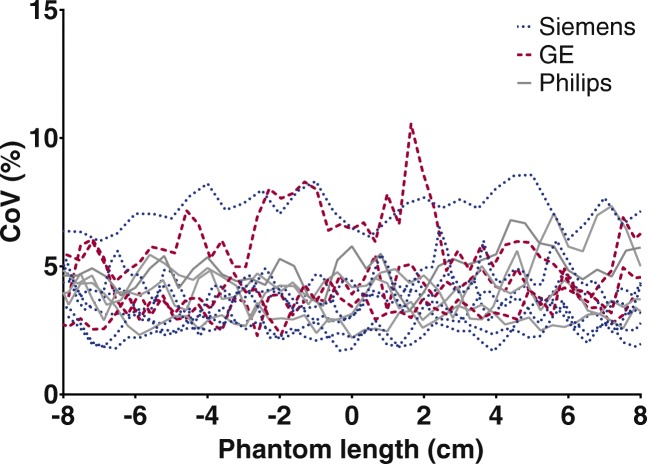
Noise across the cylindrical phantom filled with ^68^Ga, visualized as coefficient of variation (CoV)

An overview of all PET/CT systems and reconstruction settings is provided in Table 2. For local cross-calibration, most systems performed within 10% deviation of the dose calibrator (Fig. 1). The median [IQR] ratio was 0.93 [0.91–0.98] and 0.99 [0.97–1.01] for ^68^Ga and ^18^F, respectively. Two systems showed identical calibration accuracy for both isotopes (system 2 and 11), all other show a consistent underestimation for ^68^Ga. The ^68^Ga CoV in the centre of the cylindrical phantom was below 10% (Fig. 2).

**Table 2:** PET/CT reconstruction settings for phantom measurements

No.	Manufacturer	PET/CT system	Reconstruction	Iterations	Subsets	Filter size (mm)	Matrix	Voxel size (mm)	Slice thickness (mm)
1	Siemens	Biograph mCT 40 (1)	PFS + TOF	3	21	7.00	256 × 256	3.18	3
2	Siemens	Biograph mCT 40 (2)	PFS + TOF	3	21	7.00	256 × 256	3.18	3
3	Siemens	mCT 123 X3R	Back projection	–	–	5.00	200 × 200	4.07	5
4	Siemens	Biograph mCT Flow 20	PFS + TOF	2	21	5.00	200 × 200	4.07	2.027
5	GE	VCT	3D IR^†^	NS	NS	NS	128 × 128	5.47	3.27
6	GE	Discovery D690	VPFXS*	4	8	NS	192 × 192	3.65	3.27
7	Philips	Gemini TOF	BLOB-OS-TF	3	31	NS	144 × 144	4	4
8	Philips	Gemini TOF BigBore	BLOB-OS-TF	3	31	NS	144 × 144	4	4
9	Philips	Ingenuity	BLOB-OS-TF	3	31	NS	169 × 169	4	4
10	Philips	Vereos	BLOB-OS-TF	3	15	3.00	144 × 144	4	4
11	GE	Discovery 710	VPFX^§^	NS	NS	NS	256 × 256	2.73	3.27
12	Siemens	mCT 40	PFS + TOF	3	21	6.50	256 × 256	3.18	2
13	Siemens	mCT 64	PFS + TOF	3	21	6.50	256 × 256	3.18	2

TOF or *TF* = time-of-flight, *PSF* = point-spread-function, *NS* = not specified

†3D OSEM

*3D OSEM with TOF and PSF

§3D OSEM with TOF

**Fig. 3:**
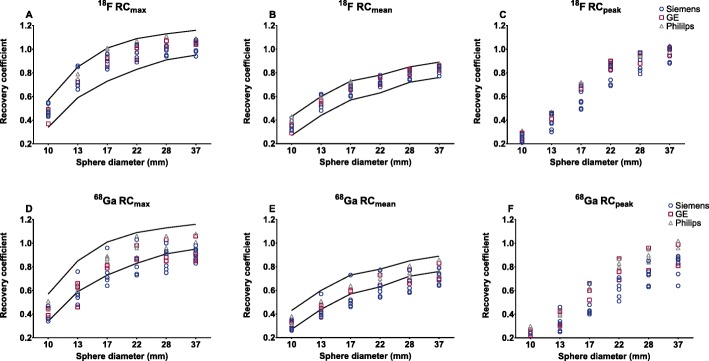
RC for ^18^F with the current EARL standards and RC of ^68^Ga. Solid lines: maximum and minimum values according to EARL limits as applicable before 2019

The ^18^F RC-curves of all PET/CT systems satisfied the current EARL specifications (Fig. 3a–c). However, for ^68^Ga the RC-curves were located around the lower limit of the EARL specifications (Figure 3d-f). In addition, ^68^Ga showed a reduced mean recovery and larger variation between PET/CT systems compared to the ^18^F. The variation for all spheres of the RC_mean,_ RC_max_ and RC_peak_ for ^18^F was 6%, 6% and 8%, respectively. For ^68^Ga, the mean range was 11%, 11% and 15% (largest variation was 19%). Furthermore, the mean RC_max_ and RC_mean_ were both 11% lower compared to the mean EARL specifications for ^18^F. The mean ^68^Ga/^18^F calibration difference within one scanner was 7% (range 1–13%).

**Fig. 4:**
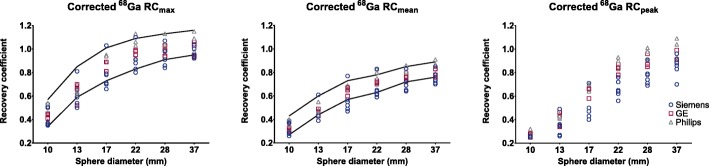
^68^Ga RC-curves corrected for the ^18^F/^68^Ga calibration mismatch according to local cross-calibration. Solid lines: maximum and minimum values according to EARL limits as applicable before 2019

After correction for the local difference between ^68^Ga/^18^F cross-calibration (Fig. 1), the ^68^Ga RC curve was within EARL limits for all but two scanners (Figure 4). The mean ^68^Ga RC_max_ and RC_mean_ were accordingly 5% lower compared to mean EARL standards.

## Discussion

In this study, quantitative ^68^Ga PET/CT performance was evaluated in a multicentre setting. In a survey across Dutch hospitals, differences in clinical acquisition and reconstruction protocols were observed, underlining the need for clinical harmonisation. Although 11 out of the 13 PET/CT systems were EARL accredited, all systems showed ^18^F recovery performance within EARL standards. For this reason, all systems were included for ^68^Ga evaluation.

The absence of local and central dose calibrator cross-calibration for ^68^Ga is a limitation in this study. This would increase local calibrator harmonisation and improves PET/CT comparability across sites. Most institutes use a long-lived (^137^Ceasium) source to assess constancy and accuracy of the dose calibrator on a daily basis, and perform actual cross-calibration with the PET/CT system at least once a year using ^18^F. Still, in all but three PET/CT systems the measured ^18^F and ^68^Ga activity concentrations were within 10% deviation from the local dose calibrator. High energy prompt gammas emitted by ^68^Ga are likely detected by the dose calibrator causing a disconcordance, yet in fewer extent by the PET system. Because of this, the dose calibrator overestimates ^68^Ga-activity, and a persistent underestimation for ^68^Ga compared to ^18^F is seen in Fig. 1. A recent study by Bailey et al. also showed an underestimation of ± 15% for ^68^Ga, which was primarily related to an inaccurate scaling factor for the dose calibrator of a specific vendor [9]. To avoid these issues, they calibrated the dose calibrator towards the PET, after verifying that the scanner has a good response for ^18^F. These results are also supported by the fact that on specific Siemens scanners (scanners 1 and 2), a traceable ^68^Germanium (^68^Ge) source was used to verify absolute PET response independent of a dose calibrator. When imaging the ^68^Ge-source, the PET/CT system did not show the same offset as was observed when imaging the ^68^Ga cross-calibration phantom (roughly a deviation of < 1% vs. 6% and 7%, respectively). For the sake of simplicity, we would suggest to correct the RC curve for the local ^68^Ga/^18^F discrepancy, as after correction for this ^68^Ga/^18^F difference (Fig. 4) all but two scanners were within EARL specifications. This correction has to be performed offline in multicentre quantitative studies. The ^68^Ga used for this study was produced either locally or by a pharmaceutical institution and was therefore not traceable to a central dose calibrator. We expect that the response between the dose calibrator and the PET-system could be uniform in future clinical ^68^Ga-PET/CT studies if a traceable (NIST) source is used to harmonise protocols between centres.

^68^Ga image noise was below 10% for all PET/CT systems which is in concordance with the EANM/EARL guidelines [3, 8]. The RC variation is larger for ^68^Ga compared to ^18^F (Fig. 3). However, ^68^Ga performance nearly reached EARL performance specifications after correction for the local ^68^Ga/^18^F ratio. Surprisingly, the RC_peak_ variation (8% and 15%) is larger in contrast to RC_max_ and RC_mean_ (both 6% and 11%) for both ^18^F and ^68^Ga, respectively. The study of Kaalep et al. showed the opposite result in RC_peak_ variation [5]. The RC_peak_ is expected to be less prone to noise compared to RC_max_; therefore, it was expected to be more comparable over all PET-systems. The difference could be explained by the fact that the standard deviation of RC_max_ and RC_peak_ are similar: 8.4% and 8.6% for ^68^Ga and 4.8% and 5.0% for ^18^F, respectively. Yet, the mean RC_peak_ value is lower; therefore, resulting in a higher CoV. Next to that, the larger ^68^Ga variation in the RC-curves compared to ^18^F is likely related to the higher positron energy of ^68^Ga and thereby revealing a lower signal-to-noise ratio. This effect is enhanced by post-reconstruction filtering. Finally, previous single-centre studies show ^68^Ga RC-curves similar [10] or somewhat better due to point spread function reconstruction [11] as observed in the current study. The EARL limits as applicable before 2019 (EARL1) are shown in Figs. 3 and 4, as all acquisitions were acquired before 2019 and therefore site-specific acquisition and reconstruction protocols are designed to meet the EARL1 limits. RC_peak_ specifications are not available for EARL1 and are therefore not shown in Figs. 3 and 4. EARL2 limits (applicable from 2019) for RC_max_ and RC_mean_ increased with ~ 25% in comparison to EARL1. We expect that the gap between ^18^F and ^68^Ga recoveries will further increase with these new limits, as already for EARL1 not all scanners agreed to EARL1 limits after ^68^Ga/^18^F correction (Fig. 4).

Based on the results, we propose to correct ^68^Ga recovery towards the ^18^F recovery to correct for the current dose calibrator deviation. We suggest, therefore, to apply the EARL acquisition and reconstruction protocol and to correct for ^68^Ga/^18^F cross-calibration mismatch. One can assume that ^68^Ga recovery is steady if ^18^F specifications of a PET-system are stable during regular yearly assessment. Unless the acquisition and reconstruction protocol is changed or major maintenance is performed to the PET/CT-system, we recommend to perform additional ^68^Ga IQ acquisitions only when regular ^18^F evaluations are deviating. An EARL accreditation programme for ^68^Ga can thus be based on the ^18^F accreditation but extended with a cross-calibration verification between ^68^Ga measured by the dose calibrator and PET/CT system only, similarly as proposed by Kaalep et al. for ^89^Zr [12]. In addition, frequent ^18^F cross-calibration acquisitions using the cylindrical phantom are advised, especially after PET/CT system maintenance.

## Conclusion

This evaluation of multicentre ^68^Ga PET/CT performance showed that ^68^Ga RCs perform at the lower limits of current ^18^F EARL standards. For practical reasons, we recommend to use the ^18^F EARL approved reconstruction settings and to correct for ^68^Ga/^18^F calibration mismatch based on local cross-calibration. Finally, we suggest to evaluate ^68^Ga PET/CT recovery performance once and repeat only when ^18^F specifications are changed.

## Data Availability

The datasets used and/or analysed during the current study are available from the corresponding author on reasonable request.

## Abbreviations

^18^F: ^18^Fluorine
^68^Ga: ^68^Gallium
^89^Zr: ^89^Zirconium
CoV: Coefficient of variation
EARL: EANM Research Ltd
IQ: Image quality
RC: Recovery coefficient
VOI: Volume-of-interest
